# Benzimidazole resistance-associated mutations improve the *in silico* dimerization of hookworm tubulin: An additional resistance mechanism

**DOI:** 10.14202/vetworld.2024.2736-2746

**Published:** 2024-12-06

**Authors:** Jan Clyden B. Tenorio, Muhammad Fikri Heikal, Alok Kafle, Prasert Saichua, Sutas Suttiprapa

**Affiliations:** 1 Tropical Medicine Graduate Program, Department of Tropical Medicine, Faculty of Medicine, Khon Kaen University, Khon Kaen, 40002, Thailand; 2 Department of Veterinary Paraclinical Sciences, College of Veterinary Medicine, University of Southern Mindanao, Kabacan 9407, Cotabato, Philippines; 3 Department of Tropical Medicine, Faculty of Medicine, Khon Kaen University, Khon Kaen, 40002, Thailand; 4 WHO Collaborating Center for Research and Control of Opisthorchiasis (Southeast Asian Liver Fluke Disease), Tropical Disease Research Center, Khon Kaen University, Khon Kaen 40002, Thailand

**Keywords:** *Ancylostoma*, anthelmintic resistance, microtubules, soil-transmitted helminths

## Abstract

**Background and Aim:**

Mutations in the β-tubulin genes of helminths confer benzimidazole (BZ) resistance by reducing the drug’s binding efficiency to the expressed protein. However, the effects of these resistance-associated mutations on tubulin dimer formation in soil-transmitted helminths remain unknown. Therefore, this study aimed to investigate the impact of these mutations on the *in silico* dimerization of hookworm α- and β-tubulins using open-source bioinformatics tools.

**Materials and Methods:**

Using AlphaFold 3, the α- and β-tubulin amino acid sequences of *Ancylostoma ceylanicum* were used to predict the structural fold of the hookworm tubulin heterodimer. The modeled complexes were subjected to several protein structure quality assurance checks. The binding free energies, overall binding affinity, dissociation constant, and interacting amino acids of the complex were determined. The dimer’s structural flexibility and motion were simulated through molecular dynamics.

**Results:**

BZ resistance-associated amino acid substitutions in the β-tubulin isotype 1 protein of hookworms altered tubulin dimerization. The E198K, E198V, and F200Y mutations conferred the strongest and most stable binding between the α and β subunits, surpassing that of the wild-type. In contrast, complexes with the Q134H and F200L mutations exhibited the opposite effect. Molecular dynamics simulations showed that wild-type and mutant tubulin dimers exhibited similar dynamic behavior, with slight deviations in those carrying the F200L and E198K mutations.

**Conclusion:**

Resistance-associated mutations in hookworms impair BZ binding to β-tubulin and enhance tubulin dimer interactions, thereby increasing the parasite’s ability to withstand treatment. Conversely, other mutations weaken these interactions, potentially compromising hookworm viability. These findings offer novel insights into helminth tubulin dimerization and provide a valuable foundation for developing anthelmintics targeting this crucial biological process.

## Introduction

Soil-transmitted helminth infections remain the most prevalent neglected tropical diseases worldwide [1, 2]. Among these helminths, hookworms are of particular public health concern due to the severity of their pathological consequences of infections [3]. Recent reports indicate that the prevalence of hookworms in endemic areas ranges from 32% to 79%, leading to 800,000 to 4 million disability-adjusted life years lost [4, 5]. These blood-sucking pathogens also cause significant infection and illness burdens in companion animals [6, 7]. To address this public and veterinary health concern, therapeutic interventions aimed at eliminating and preventing infections are currently the primary approach being implemented [8, 9]. For humans, the World Health Organization recommends prolonged administration of benzimidazole (BZ) to susceptible populations to reduce prevalence, decrease infection intensity, and prevent reinfection [10]. In animals, routine veterinary deworming is performed to achieve objectives similar to those of human interventions [11–13]. However, the repeated and extended use of these pharmacological interventions has raised concerns about the emergence of resistance to the drugs used in these programs [14, 15].

BZs are anthelmintic drugs that disrupt microtubule assembly by inhibiting tubulin dimerization through interaction with the β-tubulin subunit [16]. Resistance to these drugs is conferred by mutations in the β-tubulin gene of helminths [17]. Single-nucleotide mutations in these genes lead to amino acid substitutions that potentially alter the structure of the expressed β-tubulin, reducing the binding efficiency of BZ drug ligands [18, 19]. Canonical amino acid substitutions associated with BZ resistance include changes in positions 167 (TTC, TTT/phenylalanine → TAC, TAT/tyrosine), 198 (GAG, GAA/glutamic acid → GCG, GCA/alanine), and 200 (TTC/phenylalanine → TAC/tyrosine) [20, 21]. Several variants of these mutations have also been reported, such as Q134H, E198K, E198V, and F200L [14, 22–25]. These mutations are prevalent in *Ancylostoma* *caninum* populations across the United States and Canada and have been documented in various hookworm species across Africa, South America, the Caribbean, New Zealand, and Australia [24, 26–29]. All the aforementioned mutations have been experimentally validated as conferring resistance, either through genetic engineering of *Caenorhabditis elegans* or by inducing resistance in laboratory settings followed by genetic analysis [23, 30, 31]. The emergence of these mutations in hookworms from endemic regions poses a significant threat, as they could compromise existing control and eradication efforts targeting hookworm infections in humans and animals.

The role of BZ resistance-associated mutations in conferring resistance has been well studied and well-established *in vivo*, *in vitro*, and *in silico* in nematodes such as *C. elegans* [30, 32], *Haemonchus contortus* [33], and *Trichinella spiralis* [34], among others. However, little is known about the impact of resistance-associated mutations on normal tubulin function in soil-transmitted helminths. Experimental evidence indicates that hookworms harboring certain resistance-associated mutations that confer BZ resistance may experience decreased survival rates due to fitness costs imposed by the mutations [23, 35]. In contrast, several field studies by Evason *et al*. [22], Venkatesan *et al*. [25], and George *et al*. [36] have reported that hookworms carrying resistance-associated mutations can survive BZ treatment and continue to harbor these mutations post-treatment. The varying survival rates observed in hookworms with BZ resistance-associated mutations may be attributable to unidentified effects on tubulin function. Given the limited understanding of how these resistance-associated mutations affect tubulin dimerization in soil-transmitted helminths, this study computationally investigated the impact of these mutations on hookworm α- and β-tubulin dimerization using open-source bioinformatics tools.

## Materials and Methods

### Ethical approval

This is an *in silico* study and did not require ethical approval as this study was not based on animals or humans.

### Study period and location

This is an *in silico* study conducted during May and June 2024 using open access platforms named in the subsequent sections.

### Prediction of hookworm tubulin dimer folding through AlphaFold3

*Ancylostoma ceylanicum* was used as the model hookworm for the current *in silico* study as described by Medeiros *et al*. [23] and Furtado *et al*. [35] for assessing BZ resistance in hookworms. The UniProt server was used to retrieve the amino acid sequences of α-tubulin (UniProt ID: A0A016VPU0) and β-tubulin (UniProt ID: A0A0D6MC88) proteins from *A. ceylanicum* (https://www.uniprot.org/) [37]. BZ resistance-associated mutations occur in the β-tubulin isotype 1 gene of several hookworm species [6], making the isotype 1 β-tubulin of *A. ceylanicum* the focus of this research. Known resistance-associated non-synonymous mutations were introduced to the β-tubulin protein sequences using BioEdit Sequence Alignment Editor version 7.2.5 [38] (https://bioedit.software.informer.com/). These amino acid substitutions include Q134H, F167Y, E198A, E198K, E198V, F200Y, and F200L [14, 22–25, 39].

AlphaFold 3 was used to predict the folding of the tubulin heterodimer using the amino acid sequences of both wild-type and mutated α- and β-tubulin. AlphaFold 3 leverages artificial intelligence and extensive training on biomolecular data to predict 3D structures and interactions of biological molecules, including protein complexes [40] (https://golgi.sandbox.google.com). The amino acid sequences were uploaded to the server, and one copy of each of α- and β-tubulin subunits was modeled as separate proteins with two molecules of guanosine-5’-triphosphate (GTP) added as ligands. The addition of GTP molecules was used to simulate their role in the dynamic instability of microtubules, which depends on GTP binding and hydrolysis within each tubulin subunit [41]. No post-translational modifications were made to either tubulin subunit. The 3D structure, the per-residue distance distribution test (pIDDT), inter-residue total contact (ipTM), and per-residue total contact (pTM) scores, along with the predicted aligned error plots, were documented and scrutinized. The pIDDT, ipTM, and pTM metrics evaluate the accuracy of predicted protein structures by assessing the agreement between predicted and experimental residue distances, total contacts, and per-residue contacts [40]. All models with ipTM and pTM scores of 0.8 or higher were considered high-quality predictions and were downloaded. The.cis files from the server were further assessed using BIOVIA Discovery Studio Visualizer version 21.1.0.20298 (https://discover.3ds.com/discovery-studio-visualizer-download) and saved in pdb format for downstream applications. The folded tubulin dimers were further refined using GalaxyWEB’s GalaxyRefineComplex server. The server was employed to enhance the protein-protein complex’s interface contact and inter-protein orientation through side-chain repacking and molecular dynamics relaxation (//galaxy.seoklab.org/cgi-bin/submit.cgi?type=COMPLEX) [42].

### Quality assurance checks of the modeled dimers

The quality of modeled and refined tubulin heterodimers was assessed using various tools within the UCLA-DOE LAB, SAVES v6.0 platform (https://saves.mbi.ucla.edu/). VERIFY3D was used to assess the compatibility of the 3D protein structure with its amino acid sequence [43], with scores higher than 80% considered indicative of good-quality predictions. PROCHECK’s Ramachandran Plot was used to identify the allowed regions for the phi and psi backbones dihedral angles of the tubulin complex’s amino acid residues [44]. If >90% of the residues were in their most favored region, the modeled dimer was considered of excellent quality. In addition, the protein structure analysis server was used to identify errors in the 3D structure of proteins through statistical analysis against known protein data from databases (https://prosa.services.came.sbg.ac.at/prosa.php) [45]. Tubulin dimer models were uploaded to the server, and each subunit was individually analyzed [Accessed: 18 May 2024]. Models with z-scores below −5.0 were considered high-quality. The predicted wild-type tubulin dimer was subjected to pairwise structural alignment using the PDB Pairwise Structure Alignment Tool (https://www.rcsb.org/alignment) [Accessed: 18 May 2024], with the tubulin dimer of *Bos taurus* reported by Löwe *et al*. [46] as the template (PDB ID No.: 1JFF). The root mean square deviation (RMSD), Template modeling score (TM-score), identity, sequence length, and modeled residues were documented and evaluated.

### Tubulin Heterodimer interactions

The folded hookworm tubulin dimer’s protein-protein interactions were initially investigated using the HawkDock server (http://cadd.zju.edu.cn/hawkdock/), specifically through HawkDock’s Molecular Mechanics/Generalized Born Surface Area analysis platform to estimate the binding free energy (ΔG_bind), which reflects the thermodynamic favorability of the protein-protein interaction [47]. The previously energy-minimized pdb files were uploaded to the server, with chain A (α-tubulin) as the receptor and chain B (β-tubulin) as the ligand. The protein complex’s ΔG_bind (in kcal/mol) and the top five interacting amino acid residues, along with their corresponding binding free energies (in kcal/mol), were determined, documented, and assessed.

The overall binding affinity (ΔG) and dissociation constant (K_d) of the tubulin dimer were estimated using the PROtein binDIng enerGY prediction server (https://bianca.science.uu.nl/prodigy/). Using a protein complex’s 3D structure, this server employs intermolecular contacts and properties derived from the non-interface surface to predict its binding affinity [48]. The energy-minimized pdb files were uploaded, with chain A (α-tubulin) as Interactor 1 and chain B (β-tubulin) as Interactor 2 at a temperature of 250C. The ΔG (in kcal/mol) and K_d (in M) values of the tubulin heterodimers were documented and analyzed. In addition, the volume of the BZ binding site in the wild-type and mutated β-tubulin subunits was assessed using the CAVER Web v.1.2 server (https://loschmidt.chemi.muni.cz/caverweb/) [49]. The β-tubulin subunit was extracted from the tubulin dimer file and uploaded to the server. The predicted binding site encompassing amino acid positions associated with resistance mutations (i.e., 134, 167, 198, and 200) was selected.

### Molecular dynamics

Molecular dynamics simulations were performed using the WebGro server (https://simlab.uams.edu/index.php) to evaluate the behavior of the protein complex. The GROMOS96 43a1 force field was used in a triclinic system with simple point charge (SPC) water and a 0.15 M neutral NaCl salt model. The energy was minimized using the steep-descent algorithm for 5000 steps. The system was then equilibrated at 300 K and 1.0 bar pressure using the NVT/NPT (number of particles, volume, and temperature/number of particles, pressure, and temperature) ensemble and the Leap-frog integrator. Simulations of 50,000 ps duration were conducted with 1000 frames per simulation. The RMSD, root mean square fluctuation (RMSF), radius of gyration, and solvent-accessible surface area (SASA) plots were generated for analysis.

## Results

### AlphaFold 3 satisfactorily predicted the folding of hookworm tubulin dimers

**Figure-1:**
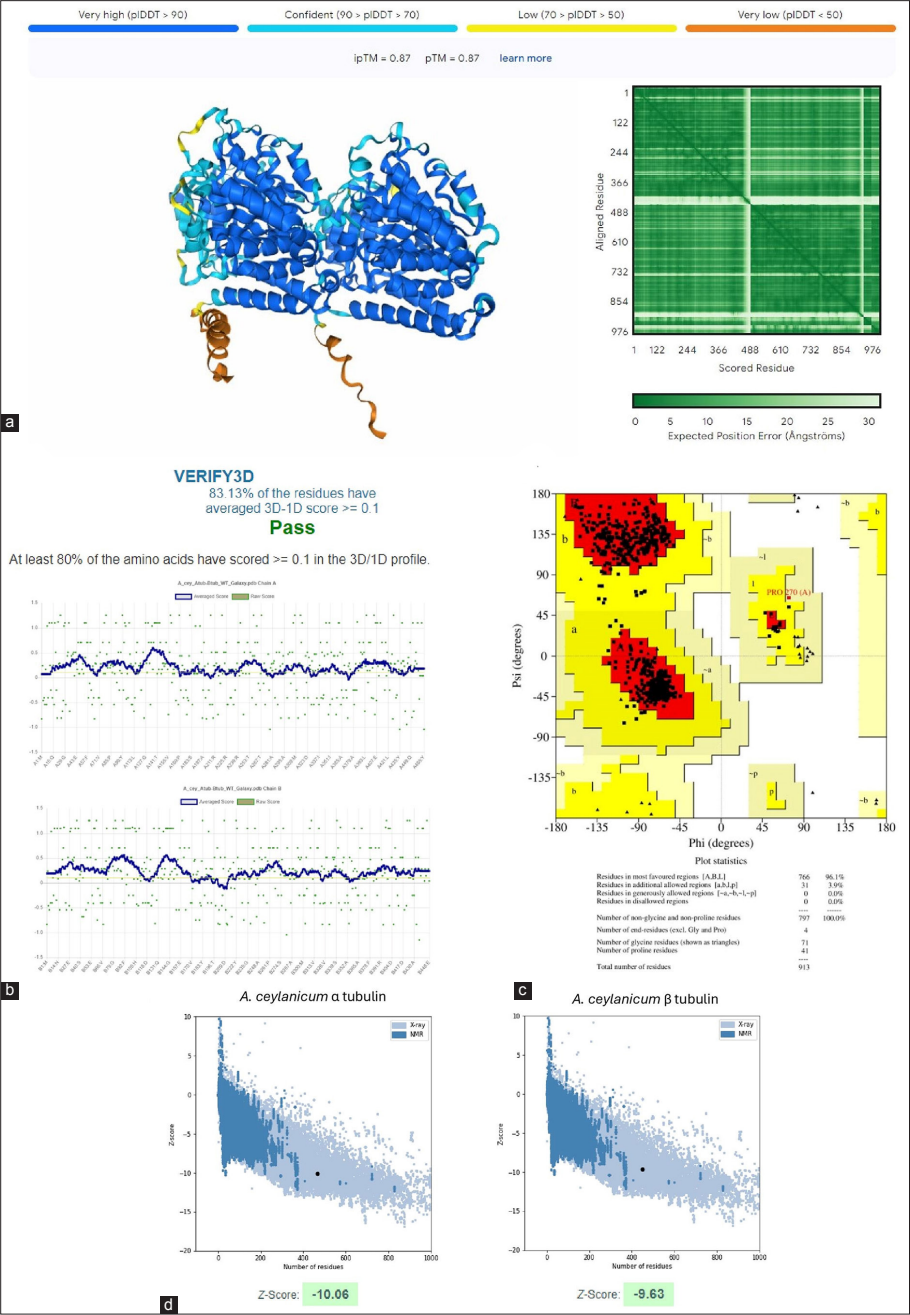
Wild-type hookworm tubulin dimer folding prediction and structural quality assurance checks. AlphaFold3 was used to predict the 3D structure of the α-β-tubulin heterodimers (a). After protein refinement and energy minimization, the modeled dimers were assessed using VERIFY3D (b), PROCHECK’s Ramachandran Plot (c), and Protein Structure Analysis (d). Overall, hookworm tubulin folding predictions from the α and β subunit amino acid sequences were deemed satisfactory and accurate.

**Figure-2:**
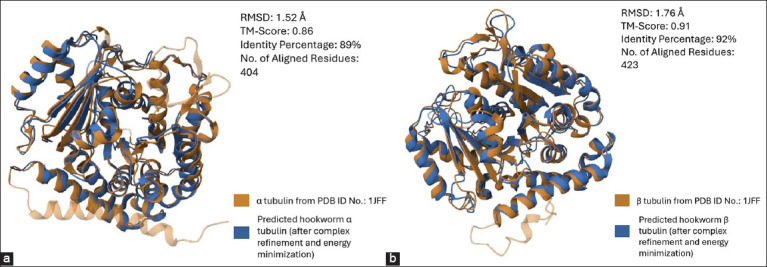
Pairwise structural alignment of the predicted folding of the hookworm tubulin α and β subunits with a previously described crystal structure of a mammalian tubulin complex (PDB ID No.: 1JFF). (a) α subunit and (b) β subunit. The analysis revealed that the modeled tubulin subunits have high structural similarity with their mammalian homologs, as determined using electron crystallography. The analysis was performed using the PDB Pairwise Structure Alignment Tool (https://www.rcsb.org/alignment).

AlphaFold 3, using its deep learning algorithms, predicted the protein-protein interactions of hookworm tubulin heterodimers based solely on the amino acid sequences of the subunits. In addition, *post hoc* complex refinement and energy minimization improved the prediction quality (Figure-1). All folded tubulin dimers (wild-type and BZ resistance-associated mutations) had pTM and ipTM scores >0.8. All folded dimers passed the VERIFY 3D assessment, with scores exceeding 80%. Furthermore, the Ramachandran plots indicated that >90% of the residues in all models were in the most favorable conformation. The dimer models were deemed to have stable and correct folding based on their z-scores below 8.0. Finally, pairwise structural alignment revealed a high degree of structural congruence between the predicted hookworm tubulin subunits and the crystal structure of their mammalian homolog (PDB ID No.: 1JFF) (Figure-2). The combined quality assurance checks from AlphaFold 3 and external verification confirmed the accuracy and reliability of the server predictions for hookworm tubulin heterodimer folding.

### BZ resistance-associated mutations affect hookworm tubulin dimerization

**Figure-3:**
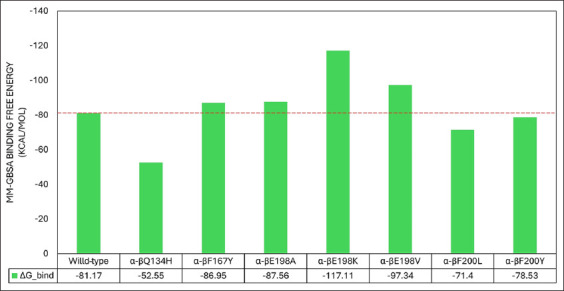
The binding free energy (ΔG_bind) of the modeled hookworm tubulin heterodimers, both wild-type and mutant. The red dashed line indicates the ΔG_bind level of the wild-type dimer. Tubulin heterodimers with the E198K and E198V mutations in the β subunit significantly increased their ΔG_bind compared to the wild-type. Interactions between the α subunit and β subunits with these mutations are more thermodynamically favorable and stable. The ΔG_bind was estimated using HawkDock’s molecular mechanics/generalized born surface area platform (http://cadd.zju.edu.cn/hawkdock/).

**Figure-4:**
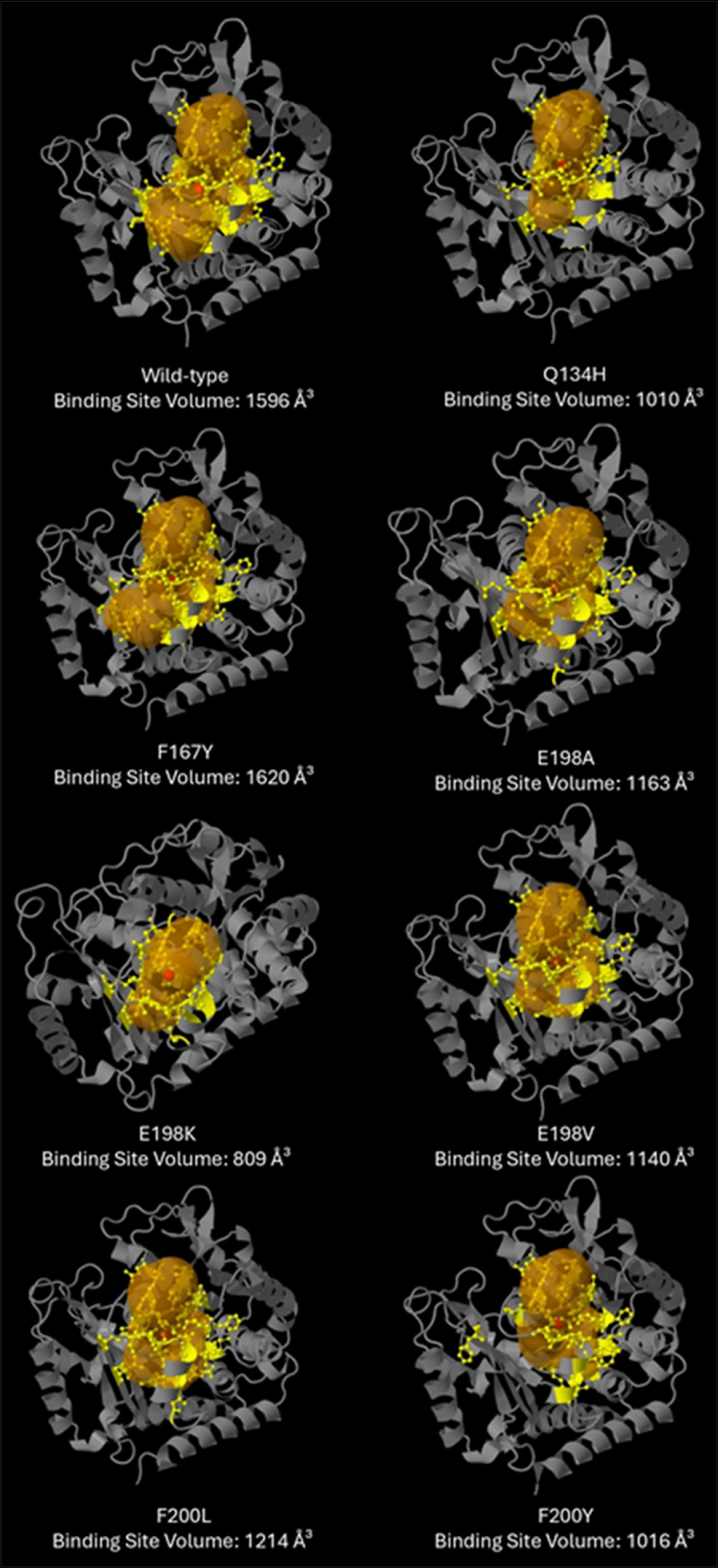
Estimated binding site pocket volume of wild-type and mutated β-tubulin subunits. Most mutations reduced the benzimidazole binding site volume. The binding site volume was estimated using CAVER Web v. 1.2 server (https://loschmidt.chemi.muni.cz/caverweb/).

BZ resistance-associated mutations in the β-tubulin isotype 1 amino acid sequences of hookworms modify binding interactions between the two tubulin subunits. Among these changes are alterations in the ΔG_bind between the subunits (Figure-3). Mutations at position 198 increased the ΔG_bind. These findings are supported by the marked increase in ΔG and reduction in K_d in dimers carrying the E198K, E198V, and F200Y mutations (Table-1). Conversely, the Q134H and F200L mutations resulted in slightly diminished ΔG but a significant increase in K_d, indicating weaker and less stable interactions. BZ resistance-associated mutations also cause changes in the interacting amino acids and their corresponding ΔG_bind between the α-and β-tubulin subunits (Table-2). These results suggest that mutations leading to amino acid substitution at position 198 may result in more stable tubulin structures, reducing their susceptibility to BZ-induced disruption and thus promoting resistance. Decreased binding values (i.e., ΔG_bind, ΔG, and K_d) caused by other mutations may weaken interactions between tubulin subunits, potentially leading to unstable microtubules, which could be harmful to the hookworm. The binding site pocket volume in the β-tubulin subunit was determined using CAVER Web v.1.2. Most mutations reduced the binding site’s volume (Figure-4), with only F167Y increasing it. A significant reduction in -tubulin volume was observed when E198A, E198K, and F200Y mutations were introduced into the β-tubulin subunits.

**Table-1:** The ΔG and K_d of the predicted hookworm tubulin dimers were estimated using the PRODIGY server (https://bianca.science.uu.nl/prodigy/).

α-β-tubulin dimer	ΔG (kcal/mol)	K_d (M)
Wild-type α-β-tubulin	−16.1	1.4e-12
α-β Q134H	−14.7	1.5e-11
α-β F167Y	−16.1	1.4e-12
α-β E198A	−14.0	5.6e-11
α-β E198K	−17.1	2.9e-13
α-β E198V	−17.0	3.3e-13
α-β F200L	−14.9	1.2e-11
α-β F200Y	−16.4	9.9e-13

ΔG=Overall binding affinity, K_d=Dissociation constant, PRODIGY: Protein binding energy prediction

**Table-2:** The top five interacting amino acids of the α and β-tubulin subunits in the folded dimers and their corresponding ΔG_bind were determined via HawkDock’s MM/GBSA (http://cadd.zju.edu.cn/hawkdock/).

α-β-tubulin dimer	α-tubulin subunit		β-tubulin subunit	
	
	Interacting amino acid	ΔG_bind (in kcal/mol)	Interacting amino acid	ΔG_bind (in kcal/mol)
Wild-type α-β-tubulin	LYS 397	−8.96	ARG 2	−7.1
	ARG 217	−8.13	LEU 436	−618
	ARG 210	−7.73	LYS 252	−6.04
	TRP 403	−7.54	ARG 251	−4.27
	ARG 398	−7.42	TRP 344	−4.09
α-β Q134H	ARG 398	−7.65	LYS 252	−7.49
	TRP 403	−7.61	ARG 2	−5.66
	ARG 210	−7.32	ASN 347	−5.06
	PHE 400	−6.78	ARG 324	−4.6
	VAL 177	−6.33	LEU 436	−4.59
α-β F167Y	ARG 398	−9.97	ARG 251	−9.31
	TRP 403	−8.22	ARG 324	−8.11
	PHE 400	−7.53	ASN 347	−6.64
	VAL 177	−7.25	ARG 2	−5.2
	ARG 210	−6.96	TRP 344	−4.76
α-β E198A	LYS 397	−8.43	ARG 251	−8.81
	TRP 403	−7.69	ARG 324	−8.75
	VAL 177	−7.44	ARG 2	−8.37
	PHE 400	−6.98	ASN 347	−5.14
	ARG 217	−6.68	TRP 311	−4.3
α-β E198K	ARG 398	−13.74	ARG 251	−9.53
	LYS 387	−9.22	ARG 324	−8.04
	ARG 217	−8.17	ARG 2	−5.62
	PHE 400	−7.8	TRP 344	−4.28
	ARG 210	−7.44	TYR 445	−4.26
α-β E198V	ARG 398	−10.02	ARG 251	−6.87
	ARG 210	−7.83	ARG 162	−5.84
	TRP 403	−7.76	ARG 2	−5.32
	PHE 400	−7.27	LEU 436	−4.25
	VAL 177	−6.62	PHE 260	−3.99
α-β F200L	ARG 217	−8.43	ARG 251	−8.64
	TRO 403	−8.14	ARG 324	−7.71
	VAL 177	−7.51	ARG 2	−4.43
	ARG 398	−7.14	TRP 344	−4.15
	PHE 400	−6.64	VAL 255	−3.64
α-β F200Y	ARG 217	−8.03	ARG 2	−10.9
	VAL 177	−7.22	ARG 324	−8.19
	PHE 400	−6.92	LYS 252	−5.09
	ARG 398	−6.81	TYR 445	−4.85
	TRP 403	−6.79	MET 433	−4.49

ΔG_bind=Binding free energy, MM/GBSA=Molecular mechanics/generalized born surface area

### Molecular dynamics simulations

**Figure-5:**
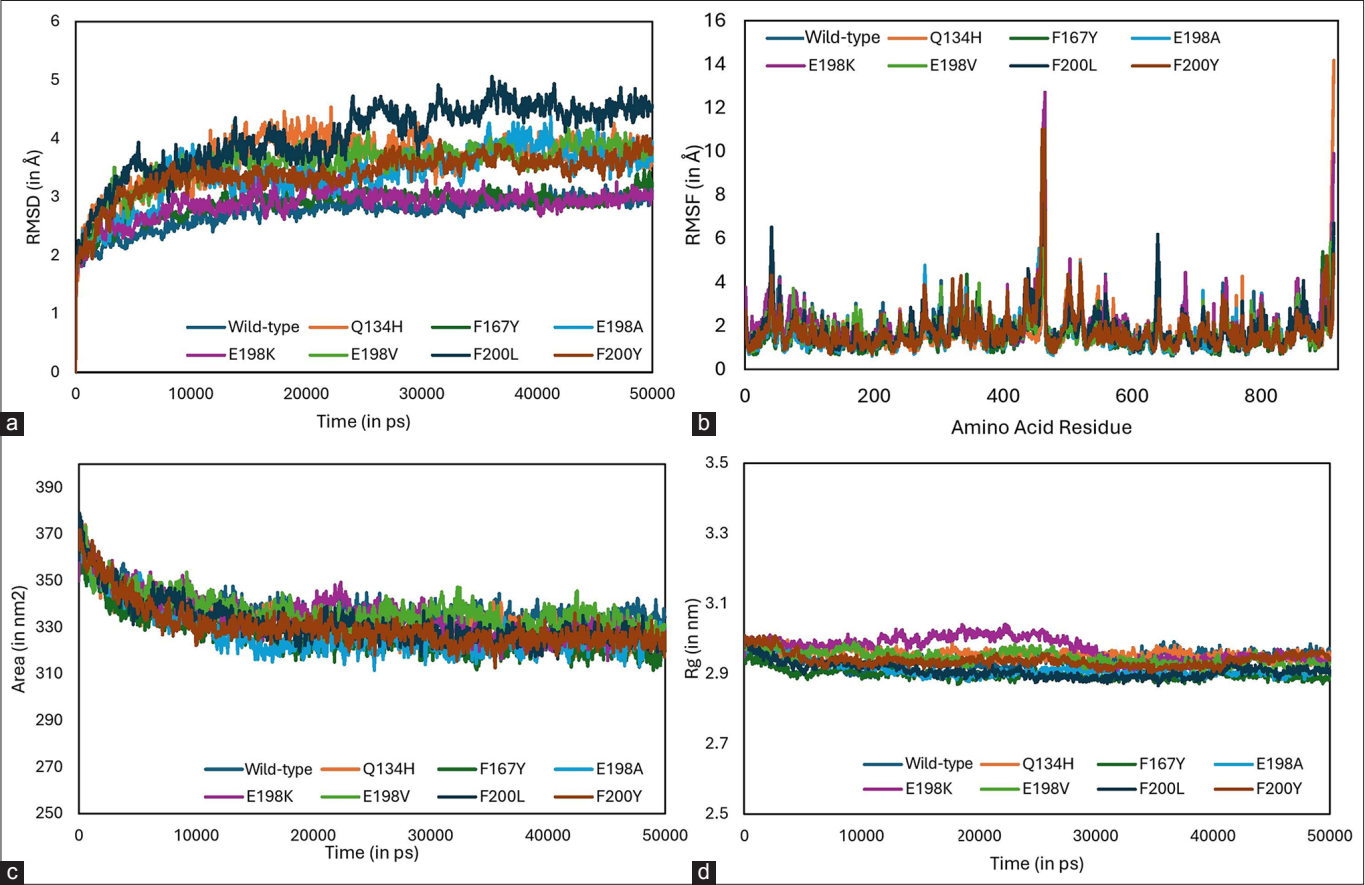
Root mean square deviation (RMSD), root mean square fluctuation (RMSF), solvent-accessible surface area (SASA), and radius of gyration plots of the wild-type and mutated tubulin dimers. (a) RMSD plot derived from 50,000 ps molecular dynamics simulations. (b) RMSF plot showing deviations per dimer residue. (c) SASA plot depicting the surface area of the tubulin dimer that is solvent accessible during the simulation period. (d) Radius of gyration plot showing the compactness of the dimer through the 50,000 ps simulation time.

Molecular dynamics simulations showed comparable behavior between folded tubulin dimers despite the presence of resistance-associated mutations. The RMSD plots indicate that dimers carrying F200Y, Q134H, and E198A mutations in the β-subunit were more stable than those carrying other mutations, including the wild type (Figure-5a). Dimers with F167Y, E198K, and E198V mutations exhibited similar dynamic behavior as the wild-type. In contrast, the dimer with the F200L mutation displayed increased RMSD deviations from 25,000 ps, suggesting that dimer binding was less stable and tended toward dissociation. This result is supported by the lowered ΔG_bind and k_d values for this mutation. The RMSF plots revealed similar trends in the flexibility of the dimer backbone relative to its constituting residues (Figure-5b), indicating that resistance-associated mutations did not significantly alter the dimer backbone’s flexibility. The increased RMSF values at residues ~440–~450 correspond to the hinge region between the dimers. A similar trend was observed in the SASA plot, in which the tubulin complex values remained relatively unchanged despite the presence of resistance-associated mutations (Figure-5c). Finally, the radius of the gyration plot showed that all mutated and wild-type complexes remained compact throughout the simulation (Figure-5d). The dimer with the E19K mutation exhibited a slight but insignificant increase in gyration from 10,000 to 30,000 ps, suggesting minor conformational changes during this simulation period. Overall, molecular dynamics simulation results suggest that tubulin dimers behave similarly, regardless of the presence of BZ resistance-associated mutations.

## Discussion

The present study aimed to assess the consequences of BZ resistance-associated mutations on the *in silico* dimerization of hookworm tubulin α and β subunits. Using *A. ceylanicum* as the model organism, the folding and interaction of tubulin heterodimers were predicted using AlphaFold 3. The folding predictions were considered accurate and reliable based on several quality assurance checks. Assessment of the dimer interactions showed that introducing resistance-associated mutations into β-tubulin altered the dimer binding properties. These mutations also modified the interacting amino acids between the α and β subunits and their corresponding binding free energies. However, molecular dynamics simulations of atomic flexibility and motion revealed that both wild-type heterodimers exhibited comparable structural flexibility and mobility. To the best of our knowledge, this is the first study on the *in silico* dimerization of hookworm tubulins with respect to BZ resistance. These results have significant implications for understanding BZ resistance in hookworms.

Our *in silico* dimerization of wild-type and mutant hookworm tubulins revealed several changes in dimer interactions due to BZ resistance-associated amino acid substitutions in the β subunit. The E198K, E198V, and F200Y mutations increased the dimer binding energies. A higher binding energy (i.e., higher ΔG_bind and ΔG, lower K_d) between the mutated α- and β-tubulin suggests a stronger interaction between these subunits. This finding is in agreement to Liu *et al*. [50], indicating that certain mutations in the α- and β-tubulin improve the stiffness, rupture force, and interface interaction energy of the formed dimer. If the mutated β-tubulin binds more tightly to its α counterpart, the BZ molecule may have difficulty binding to its target site on the β subunit [47]. This is exemplified by our results showing reduced binding pocket volume in β-tubulins with resistance-associated mutations. Reduced binding site volume and tighter interaction between tubulin subunits may confer resistance by hindering access to BZ drugs whose binding site is buried within the β subunit [51]. Moreover, stronger interactions between the subunits can prevent the curving of tubulin conformation, as observed in crystal structures bound to colchicine [52]. Therefore, increased tubulin subunit binding may contribute to BZ resistance. This notion is supported by a laboratory study conducted by Dilks *et al*. [30], indicating that E198V, E198K, F167Y, and F200Y conferred marked resistance in *C. elegans* to fenbendazole and albendazole. Furthermore, *in silico* docking studies by Jones *et al*. [18], Aguayo-Ortiz *et al*. [33], and Jones *et al*. [53] have shown that mutated tubulins with amino acid shifts at position 198 exhibit reduced binding affinity with BZs in *Trichuris trichiura*, *Ancylostoma duodenale*, *Ascaris lumbricoides*, and *H. contortus*. The increase in binding site volume resulting from the F167Y mutation may result in structural changes in the tubulin dimer, affecting its stability and interactions with other components of the microtubules [19]. Thus, BZ resistance-associated mutations enhance the hookworm’s capacity to resist treatment by strengthening and stabilizing tubulin dimer interactions, thereby hindering BZ binding to the β-tubulin subunit.

On the other hand, the Q134H and F200L mutations reduced the predicted binding energies of tubulin dimers. Low binding energies indicate weaker interaction between the mutated β- and wild-type α-tubulin, potentially resulting in a less favorable microtubule structure that is prone to disassembly and thus detrimental to the hookworm [54, 55]. This notion is supported by a laboratory study conducted by Medeiros *et al*. [23] and Pallotto *et al*. [32], showing that acquiring the resistance phenotype due to these mutations in helminths is associated with the cost of survival. Potential alterations in tubulin heterodimer binding dynamics due to resistance-associated mutations have also been reported in a study involving anticancer drugs. Natarajan and Senapati [56] reported that mutations in T237I, R282Q, and Q292E mutations in mammalian β-tubulin can modulate tubulin dimerization through allosteric changes in the N domain, which is involved in microtubule assembly. In addition, mutations in the α-tubulin (e.g., W407X, G43V, T145P, and A383T) resulted in reduced hydrogen bonding with its β counterpart and significant deviations in RMSD and RMSF values, leading to tubulin heterocomplex instability [57]. These findings, along with our results, indicate that mutations in either the β- or α-tubulin subunits confer binding alterations that affect tubulin dimer formation and subsequent microtubule assembly, which can either assist in drug resistance or be detrimental to the parasite.

Recent advancements in protein folding prediction and protein-protein interaction modeling represent a welcome development for anthelmintic resistance studies, particularly those involving BZs. Using deep learning algorithms trained on data from protein databases, AlphaFold 3 reliably and accurately predicted the dimerization of hookworm α- and β-tubulins. The crystal structures of hookworm α-and β-tubulin subunits and their dimerized conformations have yet to be elucidated by crystallography or spectroscopy. However, our structural alignment comparison with the mammalian tubulin crystal structure showed that our fold predictions from hookworm sequences had a remarkably close structural resemblance. The results of our external quality assurance checks further support this finding. The lack of available tubulin crystal structures from soil-transmitted helminths has hindered the study of BZ resistance, which should be addressed urgently. In addition, the ability to conduct *in silico* dimerization of hookworm tubulins with relative ease provides further insights into previously reported β-tubulin-BZ docking studies by Jones *et al*. [18], Aguayo-Ortiz *et al*. [33], and Jones *et al*. [53]. However, our dimerization model does not include the docked BZ ligand because the AlphaFold 3 server cannot currently incorporate such molecules in its predictions [58]. Future *in silico* studies should investigate the dimerization of hookworm tubulins in the presence of bound BZ ligands to gain a deeper understanding of the mechanism of action of BZ in disrupting tubulin function. Nevertheless, our research highlights the use of open-source platforms in the *in silico* study of drug resistance, presenting a free pipeline that is accessible to those without high-end computers or costly software.

## Conclusion

BZ resistance-associated amino acid substitutions in the β-tubulin isotype 1 protein of hookworms induce *in silico* alterations in the tubulin heterodimer interaction. The AlphaFold 3 models of hookworm tubulin heterodimers generated in this study were highly accurate and reliable. Mutations at positions 198 and 200 (i.e., E198K, E198V, and F200Y) led to stronger interactions between the α and β subunits resulting in more stable tubulin dimers and assisting in BZ resistance. Conversely, the Q134H and F200L mutations weakened these interactions. Despite these binding alterations, the structural flexibility and motion dynamics of both wild-type and mutant tubulin heterodimers were not significantly different – mutated tubulin complexes behaved similarly to their wild-type counterparts. In addition to hindering BZ binding to the β-tubulin subunit, resistance-associated mutations strengthen and stabilize tubulin dimer interactions. In contrast, other mutations weaken these interactions, which could be harmful to the hookworm. Our results provide novel insights into helminth tubulin dimerization, which may be useful for drug design and the discovery of new anthelmintics. These findings should be further validated through *in vitro* and *in vivo* studies.

## Data Availability

The data generated in this study can be made available from the corresponding author upon a request.

## Authors’ Contributions

JCBT: Conceptualization, Writing-original draft preparation, and formal analysis. MFH and AK: Writing-review and editing and formal analysis. PS: Writing-review and editing, and supervision. SS: Conceptualization, writing-review and editing, and supervision. All authors have read and approved the final manuscript.
